# Response to Letter to the Editor: “IGSF1 Deficiency Results in Human and Murine Somatotrope Neurosecretory Hyperfunction”

**DOI:** 10.1210/clinem/dgaa147

**Published:** 2020-03-24

**Authors:** Sjoerd D Joustra, Ferdinand Roelfsema, A S Paul van Trotsenburg, Harald J Schneider, Robert P Kosilek, Herman M Kroon, John G Logan, Natalie C Butterfield, Xiang Zhou, Chirine Toufaily, Beata Bak, Marc-Olivier Turgeon, Emilie Brûlé, Frederik J Steyn, Mark Gurnell, Olympia Koulouri, Paul Le Tissier, Pierre Fontanaud, J H Duncan Bassett, Graham R Williams, Wilma Oostdijk, Jan M Wit, Alberto M Pereira, Nienke R Biermasz, Daniel J Bernard, Nadia Schoenmakers

**Affiliations:** 1 Department of Medicine, Division of Endocrinology, Leiden University Medical Center, Leiden, Netherlands; 2 Department of Pediatrics, Leiden University Medical Center, Leiden, Netherlands; 3 Emma Children’s Hospital, Amsterdam UMC, University of Amsterdam, Pediatric Endocrinology, Amsterdam, Netherlands; 4 Department of Endocrinology, Ludwig-Maximilians University, Munich, Germany; 5 Department of Radiology, Leiden University Medical Center, Leiden, Netherlands; 6 Molecular Endocrinology Laboratory, Department of Medicine, Imperial College London, London, UK; 7 Department of Pharmacology and Therapeutics, McGill University, Montreal, Quebec, Canada; 8 The University of Queensland Centre for Clinical Research, Brisbane, Australia; 9 University of Cambridge Metabolic Research Laboratories, Wellcome Trust-Medical Research Council Institute of Metabolic Science, Addenbrooke’s Hospital, Cambridge, UK; 10 Centre for Integrative Physiology, University of Edinburgh, Edinburgh, UK; 11 CNRS, Institut de Génomique Fonctionnelle, INSERM, and Université de Montpellier, Montpellier, France

We thank Dr Faucz and colleagues for sharing their hypothesis that hypothyroidism, rather than growth hormone (GH) excess, may be the main driver for the acromegaloid facies observed in our patients with immunoglobulin superfamily member 1 (IGSF1) deficiency. This is a valid idea and one we gave careful consideration in the course of our study. Significantly, the majority (two-thirds) of affected participants received thyroxine replacement from shortly after birth. However, the presence or absence of acromegalic facies did not differ between untreated individuals and those on long-term levothyroxine at the time of facial photography.

Although it is true that hypothyroidism may cause coarse facies, patients with IGSF1 deficiency typically have mild to moderate hypothyroidism, especially when compared with the more severe thyroid hormone deficits that occur in primary hypothyroidism. In our clinical experience, the facies we observe in some of our patients with IGSF1 deficiency are not found in the context of biochemically comparable congenital hypothyroidism due to other etiologies. Additionally, some individuals with IGSF1 deficiency are not diagnosed with central hypothyroidism until late into adulthood, possibly due to preservation of circulating triiodothyronine levels, which mitigate clinical and metabolic manifestations of hypothyroidism (1). Adult height is also maintained in IGSF1 deficiency, arguing against significant skeletal hypothyroidism (2).

Clinical evaluation of our patients with IGSF1 deficiency revealed subtle facial coarsening without facial puffiness. Given the challenges in quantifying this impression objectively, we used facial image analysis to compare our patients’ facial characteristics with those of participants with confirmed acromegaly and with control participants. In the context of elevated median insulin-like growth factor 1 levels, increased GH secretion, and with other supportive clinical features (increased finger soft tissue thickness), we contend that an effect of excess GH remains a plausible explanation of our findings. The increased skeletal growth and organomegaly in *Igsf1*-deficient mice further support this contention. Nevertheless, we agree that further studies are required to investigate the molecular basis for our observations. The complex endocrinopathy seen in patients who are IGSF1 deficient mandates that the interpretation of their clinical phenotypes must derive from a consideration of the potential effects of multiple hormone abnormalities.

## Abbreviations

GH: growth hormone
IGSF1: immunoglobulin superfamily member 1

## Additional Information

***Disclosure Summary:*** The authors have nothing to disclose.
